# Retooling Microbiome Engineering for a Sustainable Future

**DOI:** 10.1128/msystems.00925-21

**Published:** 2021-08-31

**Authors:** Christopher E. Lawson

**Affiliations:** a Department of Chemical Engineering and Applied Chemistry, University of Toronto, Toronto, Ontario, Canada

**Keywords:** automation, machine learning, microbiome engineering, synthetic biology, systems biology

## Abstract

Microbial communities (microbiomes) have been harnessed in biotechnology applications such as wastewater treatment and bioremediation for over a century. Traditionally, engineering approaches have focused on shaping the environment to steer microbiome function versus direct manipulation of the microbiome’s metabolic network. While these selection-based approaches have proven to be invaluable for guiding bioprocess engineering, they do not enable the precise manipulation and control of microbiomes required for unlocking their full potential. Over the past 2 decades, systems biology has revolutionized our understanding of the metabolic networks driving microbiome processes, and more recently genetic engineering tools have started to emerge for nonmodel microorganisms and microbiomes. In this commentary, I discuss how systems biology approaches are being used to generate actionable understanding of microbiome functions in engineered ecosystems. I also highlight how integrating synthetic biology, automation, and machine learning can accelerate microbiome engineering to meet the sustainability challenges of the future.

## COMMENTARY

Microbial communities (microbiomes) are multicellular chemical factories that drive biogeochemical mass and energy transformations on Earth and have been harnessed as biotechnologies to clean up environmental pollutants and produce renewable energy. They are useful because they can efficiently perform complex metabolic tasks through division of labor, catalyze diverse chemical reactions by tapping into an enormous array of evolved protein functions, and remain stable under harsh or dynamic conditions due to intrinsic functional degeneracy. However, engineering of microbiomes to achieve a specified function remains challenging; most microbes, proteins, and their interactions in microbiomes are poorly characterized, and tools to predict and manipulate their function are lacking. The sustainability challenges of the coming decades will demand retooling of microbiomes for greater precision and control, for example, rewiring microbiome metabolism to produce chemicals and materials from renewable feedstocks or to achieve zero waste through materials recycling. This commentary discusses how systems biology approaches are being used to produce actionable understanding of microbiome metabolism and interactions and how integrating approaches from synthetic biology, automation, and machine learning will transform our ability to manipulate microbiome functions for biomanufacturing, resource recovery, and beyond.

Systems biology approaches have provided an opportunity to study the metabolic networks and microbial interactions underpinning microbiome function at a molecular level. This involves integrating basic biological information (e.g., biomass growth, substrate uptake, metabolite production) with different multi-omic analyses that lie on a spectrum of what might be happening (e.g., metagenome) to what is actually happening (e.g., metafluxome or metaphenome). To date, most microbiome studies have focused on the former, generating rich metagenomic data sets that can be used to recover tens to thousands of draft or complete genomes (i.e., metagenome-assembled genomes [MAGs]) of uncultivated microbes from complex communities (1, 2). These MAGs act as an essential starting point for reconstructing the metabolism of microbes in a community and as scaffolds for mapping RNA and proteins to examine metabolic activities and potential interactions. For example, metabolic reconstruction combined with genome-centric metatranscriptomic analysis identified the functional role of several poorly understood heterotrophic bacteria in anaerobic-ammonium oxidizing (anammox) bioreactors, including putative metabolite exchanges that improve nitrogen removal from wastewater (3). Similar methods have also revealed key microbes, pathways, and metabolic handoffs driving lignocellulosic waste valorization to medium-chain fatty acids, providing insights on how microbiome function could be optimized to improve product yields (4). While these studies highlight the value of sequencing tools for generating hypotheses on microbiome metabolism and interactions, the resulting information remains limited for engineering interventions unless it can be tested. This can in part be accomplished by coupling multi-omic methods with perturbation experiments or time-series analyses that uncover key microbiome properties, such as niche types and substrate preferences among community members in lipid-accumulating microbiomes (5). However, approaches to quantitatively predict mass and energy flows through microbiome metabolic networks from multi-omic information are ultimately needed to rationally design microbiome functions.

One of the largest challenges for microbiome engineering is to predict how a microbiome’s metabolic potential under a given environmental condition translates into emergent functions (phenotypes) at the individual and community scale. This requires new tools that move beyond sequencing approaches to quantify *in situ* metabolic rates and phenotypes in microbial communities at greater resolution. Stable isotope-based metabolomics and metaproteomics coupled to physiological experiments provide an opportunity to quantify microbiome metabolic interactions and fluxes. These approaches are increasingly being applied to nonmodel microorganisms that play important roles in microbiomes. For example, time-series ^13^C and ^2^H isotope tracing, metabolomics, and metabolic flux analysis (MFA) were used to quantify fluxes in the anammox bacterium “*Candidatus* Kuenenia stuttgartiensis” (6). This validated genome predictions of key central carbon pathways and revealed several instances where genomic predictions were not supported by *in vivo* metabolic fluxes, such as the operation of an incomplete oxidative tricarboxylic acid (TCA) cycle for alpha-ketoglutarate synthesis and the inability to use acetate as a carbon source (6). Similarly, these approaches have experimentally tested substrate utilization patterns and metabolic fluxes predicted by genome-scale models for the anaerobic fungus Neocallimastix lanati (7) and the nitrite-oxidizing bacterium Nitrospira moscoviensis (8), improving model prediction of their metabolic activities. Given the importance of metabolism to microbiome engineering, more widespread application of quantitative flux analysis to nonmodel microbes and further extension to microbial communities (see references 9 and 10) will accelerate efforts to discover basic principles for designing microbiomes.

Beyond understanding microbiome metabolism, improving our ability to precisely manipulate it for a desired outcome is critical for advancing microbiome engineering. While microbiome engineering has traditionally relied on selection (e.g., manipulation of environmental variables) to steer microbiomes toward a desired function, this approach results in self-assembled communities with limited control over specific metabolic processes. Moving forward, the development of genetically modified microbiomes that have tailored membership could transform our ability to design and experimentally interrogate microbiome functions, for example, developing genetically modified anaerobic digestion (11) or microbial electrosynthesis (12) microbiomes to recover valuable chemicals from waste resources or carbon dioxide, respectively. This could be achieved by combining predefined axenic or enrichment cultures that contain genetically modified organisms (i.e., synthetic microbiomes) or emerging approaches that introduce desired traits into self-assembled populations via horizontal gene transfer (13) together with selective removal of unwanted species via bacteriophages (14). While the assembly of synthetic microbiomes is potentially more controllable, an unanswered question is whether they can be designed to recreate the functional redundancy (or degeneracy) offered by self-assembled microbiomes (15). This could potentially be achieved by combining enrichment cultures of different functional guilds that already contain the necessary strain diversity; however, this will require further experimental investigation. Regardless of the approach taken, domestication of nonmodel microbes remains challenging due to host barriers, such as restriction-modification systems (16), and the stability of genetic systems and engineered organisms *in situ*. Moreover, the design landscape of possible microbiome genetic manipulations and environmental conditions is enormous even for “simple” two-member communities and will therefore require a systematic approach to traverse and find an optimal solution in a reasonable time frame.

**FIG 1:**
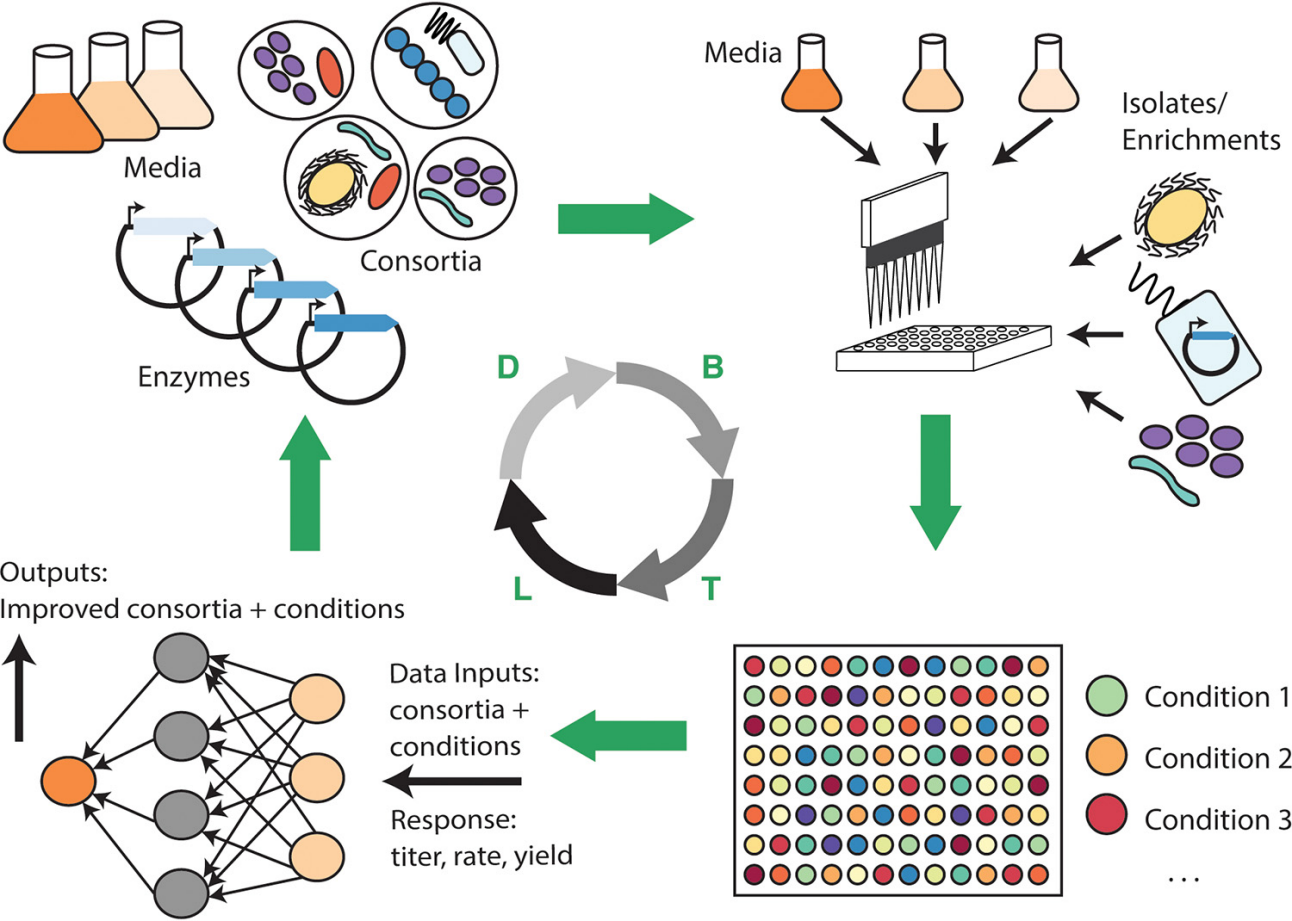
A design-build-test-learn (DBTL) cycle for engineering microbiomes driven by machine learning. Design parameters could include different media, consortia, and/or recombinant enzymes that are built using automated platforms (e.g., liquid-handling robots or microfluidics) and tested using high-throughput bioanalytical methods (e.g., fluorescence measurement or high-throughput mass spectrometry) to generate large data sets. Resulting data are used to train machine learning algorithms that accurately predict new designs that improve the desired outcome (e.g., titer, rate, yield).

To accelerate the systematic design of microbiomes, recent advances in automation, genome editing, high-throughput bioanalysis, and machine learning should be integrated to rapidly develop and test microbiomes for specific capabilities through iterative design-build-test-learn (DBTL) cycles (17) (Fig. 1). Critical to this cycle is the ability to generate large amounts of data (i.e., building and testing of hundreds to millions of unique designs) that can be leveraged to accurately predict designs that improve the desired function. For high-throughput data generation, laboratory automation, including liquid-handling robots, microfluidics, and microbioreactors, will be essential to speed up key tasks, such as isolations, bottom-up microbiome assembly, phenotype screening, genetic transformations, and fermentations. For example, droplet microfluidics was used to rapidly construct and screen ∼100,000 synthetic soil microbiomes per day (18). Such large data sets can be used to train models that learn relationships between inputs (community composition, protein levels, promoters, etc.) and outputs (chemical titer, yields, rates, etc.), and recommend new designs for testing that have an increased chance of improving outputs in subsequent cycles. Indeed, data-driven design approaches are already gaining traction for metabolic engineering of individual microbes (19) and have recently been used to assemble synthetic human gut microbiomes for improved butyrate production (20). However, many challenges still remain, particularly expanding the flexibility of screening platforms to examine more complex variables (e.g., spatial structure) and scaling up optimal solutions found in the lab to industrial applications. As the ability to perform larger and more sophisticated microbiome screening efforts coupled to machine learning increases, future studies should not only improve our ability to design microbiomes to specification but also discover novel patterns and interactions in large data sets that can be followed up with deeper multi-omic investigations for validation and elucidation.

**FIG 2:**
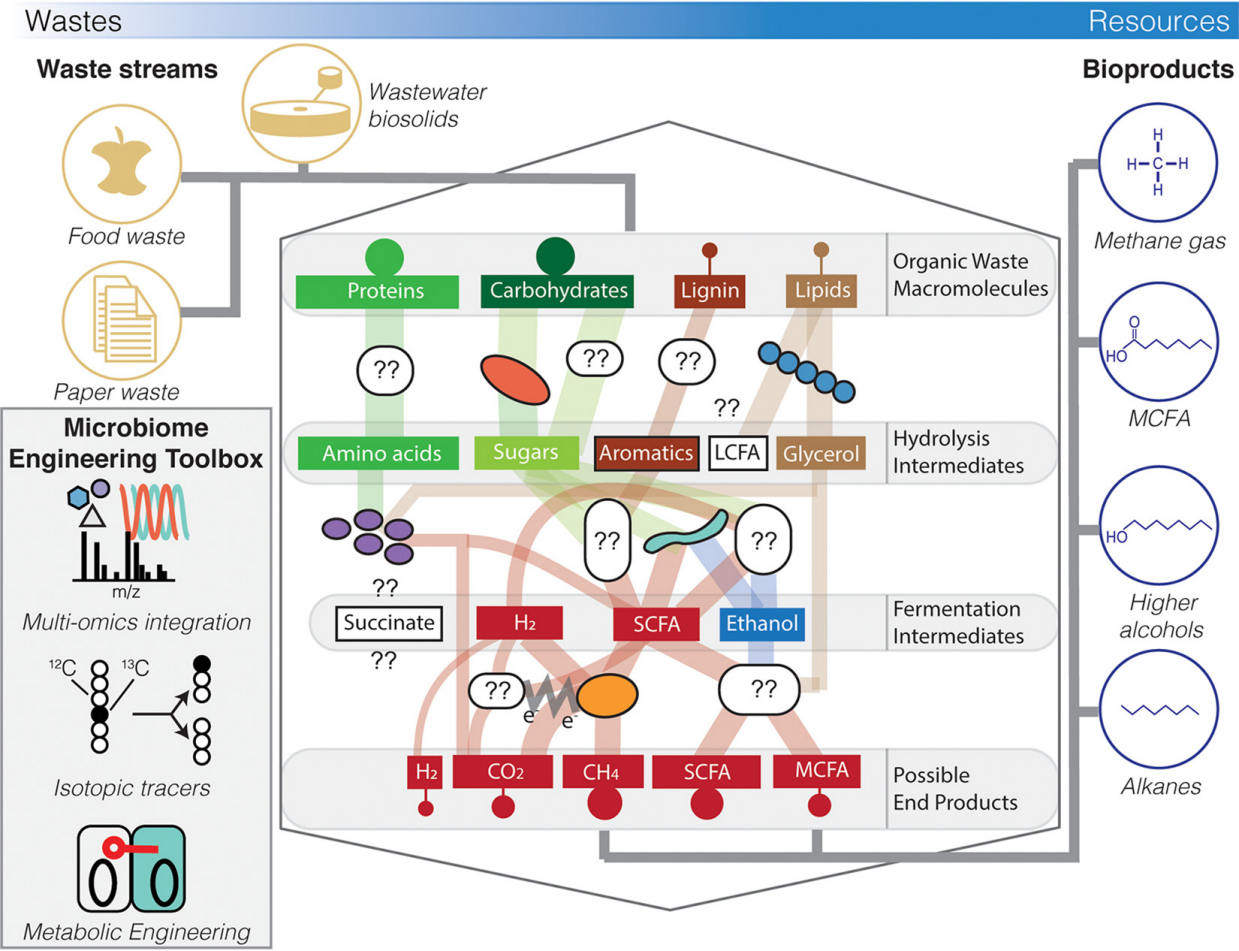
Rewiring anaerobic digestion to produce higher-value products beyond biogas (e.g., medium-chain oleochemicals) as a model system for microbiome engineering. Anaerobic digestion microbiomes can be rewired using approaches outlined in Fig. 1 and the microbiome engineering toolbox to (i) discover novel microbes, microbial physiologies, and ecological design principles driving microbiome metabolism; (ii) quantify *in situ* microbiome metabolic network activity and metabolic fluxes; and (iii) manipulate microbiome metabolism to recover valuable products from organic wastes, enabling a circular economy. White ovals indicate unknown microbes to be discovered; white rectangles indicate metabolites not connected to the microbiome metabolic network. LCFA, long-chain fatty acids (C_13_ to C_21_); MCFA, medium-chain fatty acids (C_6_ to C_12_); SCFA, short-chain fatty acids (C_2_ to C_5_).

Given the enormous complexity of microbiome interactions and the fact that the vast majority of microbes still remain poorly characterized and genetically intractable, realizing the precise engineering of microbiomes for biomanufacturing, resource recovery, and other applications will require a strategic and collaborative effort. The development and testing of simplified synthetic microbiomes provide a tractable strategy for refining methods and learning basic design rules that can be subsequently tested in more complex systems. An example model system that is particularly attractive for synthetic microbiome development is anaerobic digestion (Fig. 2), which has a wealth of previous microbiology knowledge from decades of research and can be more readily controlled in bioreactors. Our research group endeavors to rewire anaerobic digestion metabolism by integrating systems and synthetic biology, automation, and machine learning to produce high-value chemicals from wastes and renewable feedstocks. This has the potential to transform societies’ wastes into resources and offset greenhouse gas emissions through sustainable biomanufacturing. However, achieving these goals quickly will also require collaboration among diverse investigators (e.g., engineers, microbiologists, and data scientists) who coordinate microbiome engineering tool development based on open science principles. In particular, community-wide initiatives are needed to archive microbial isolate and enrichment cultures, database large phenotypic data sets from screening efforts, and extend genome editing capabilities for nonmodel organisms. This will improve data and method accessibility and accelerate tool development, catalyzing the retooling of microbiome engineering to achieve a more sustainable future.
